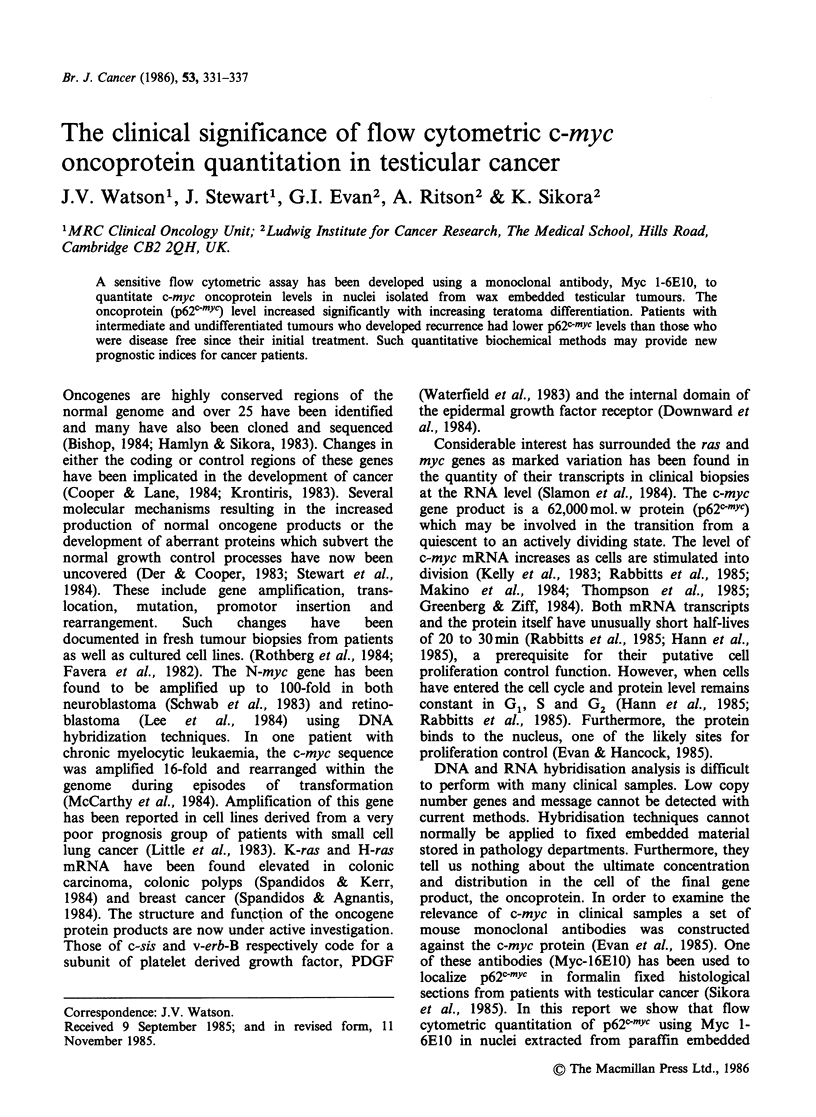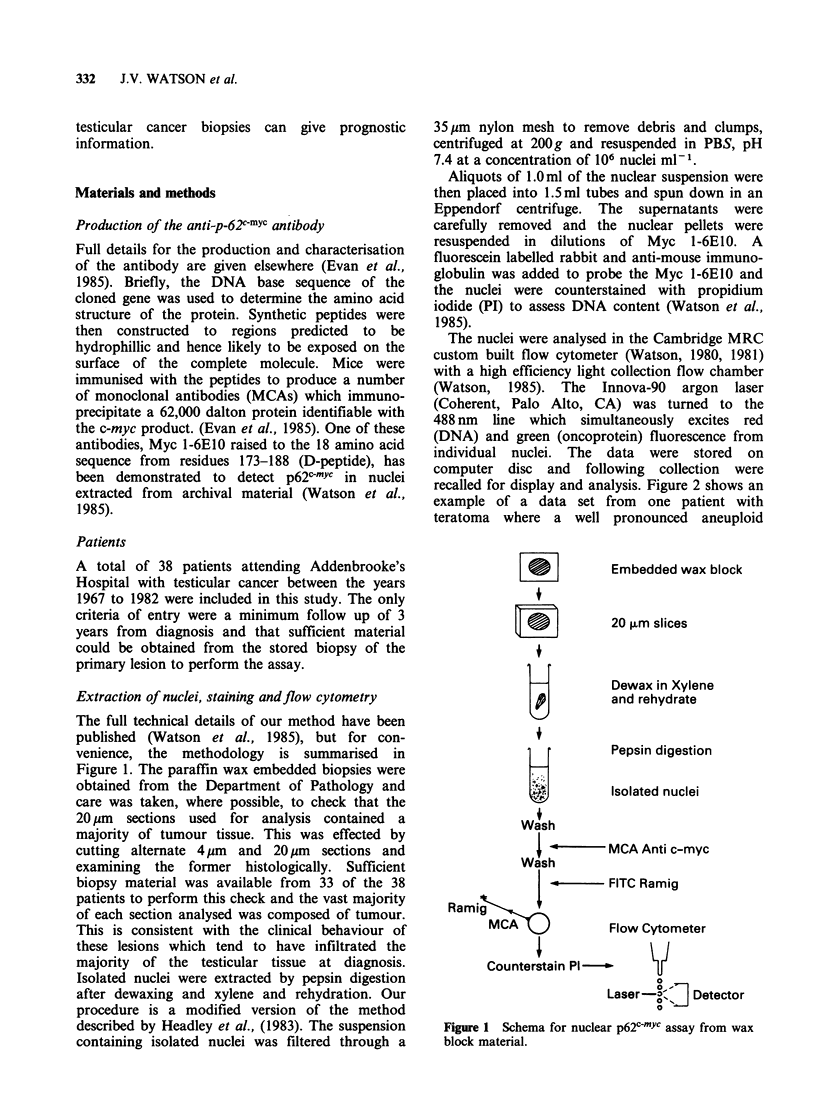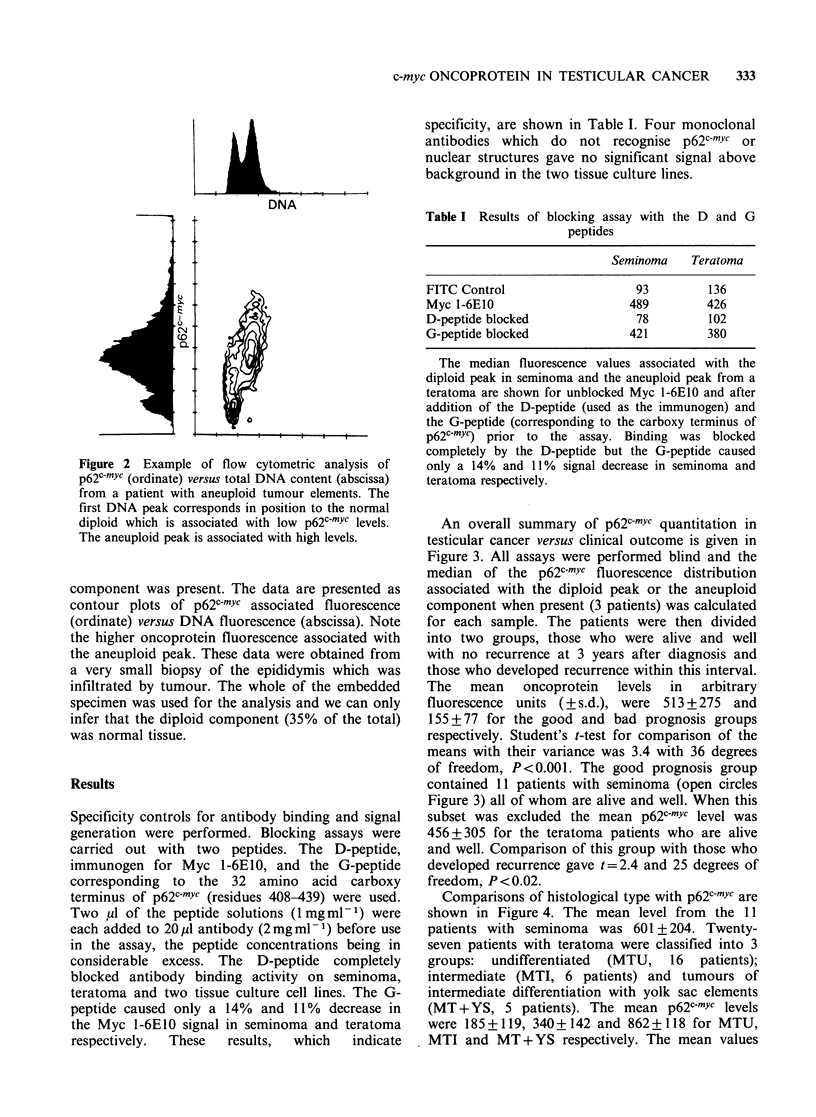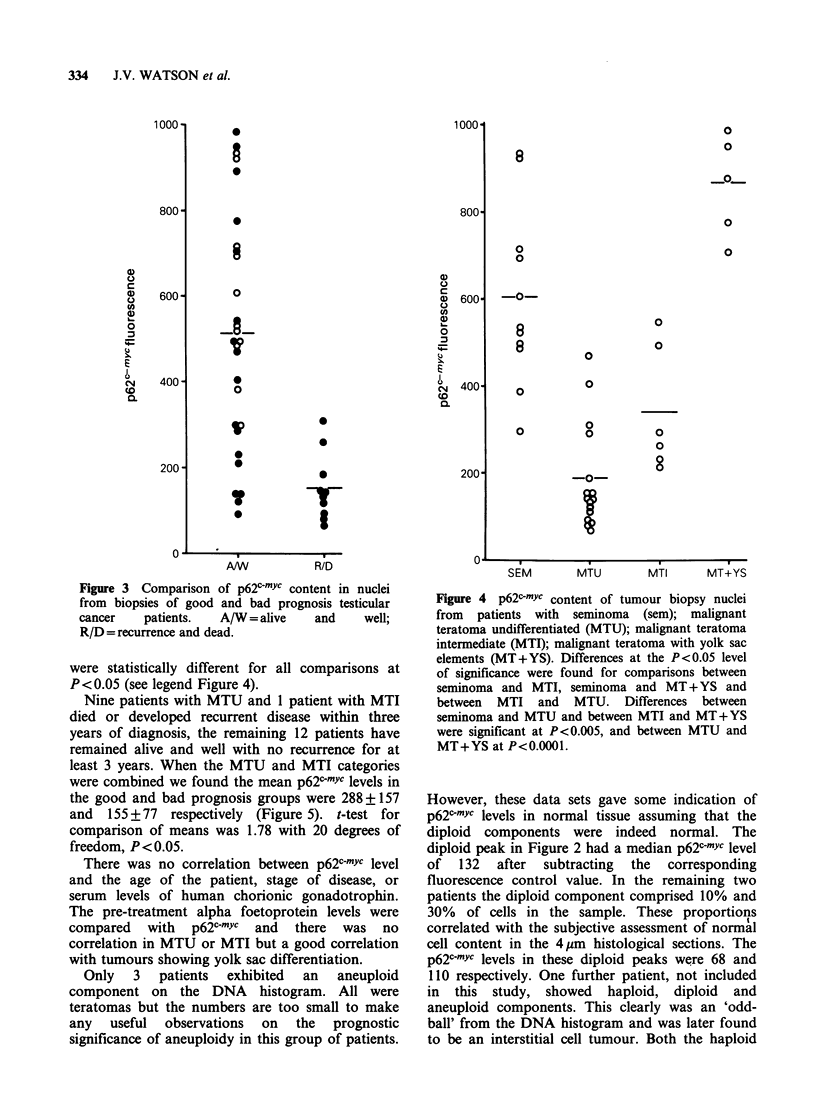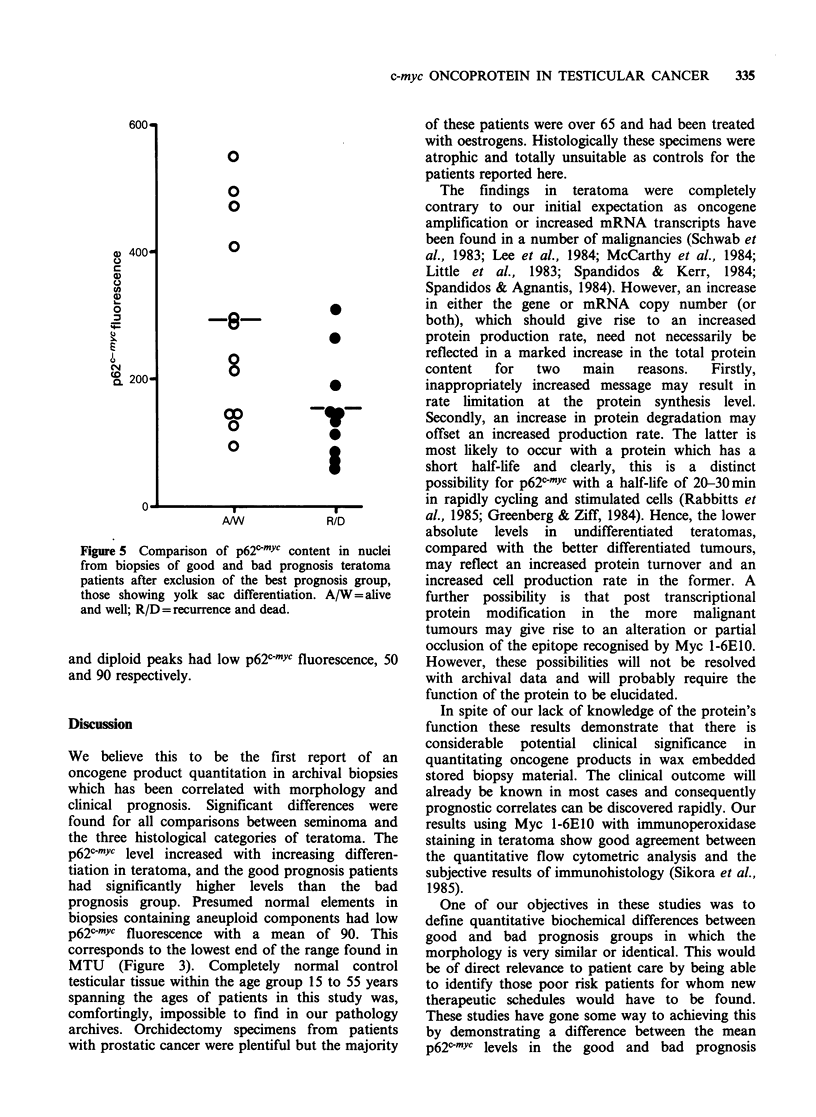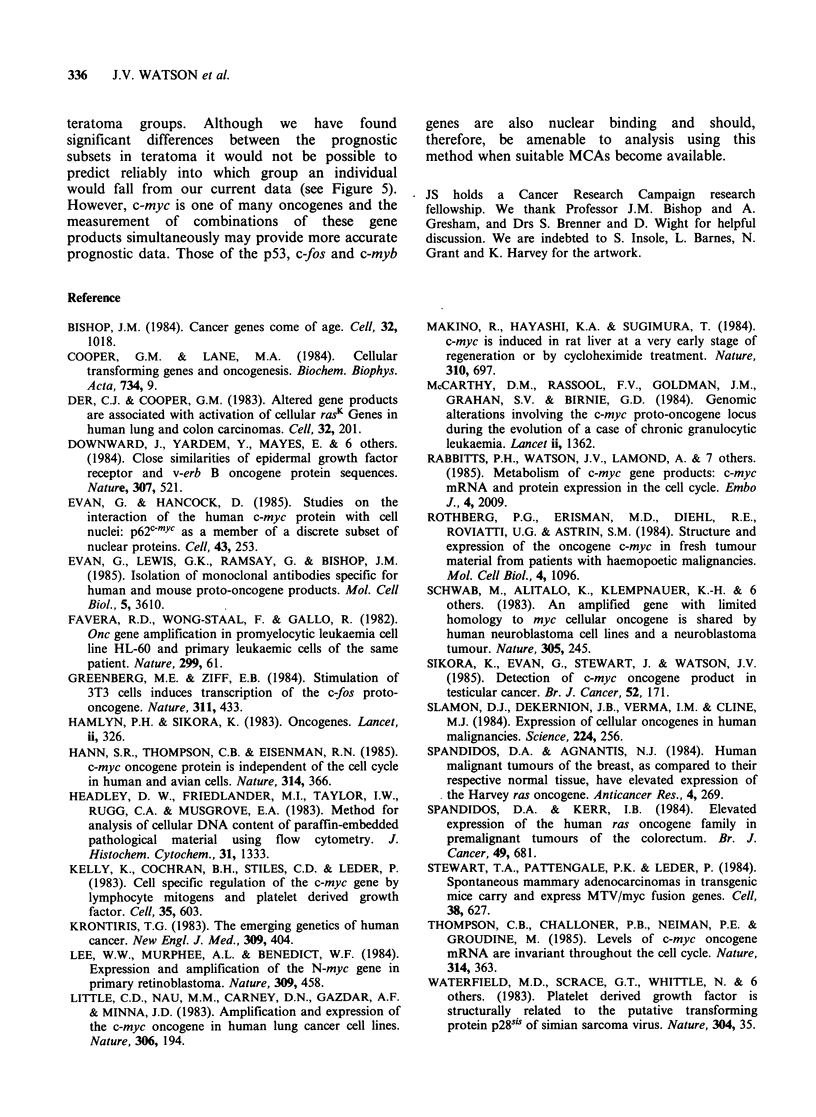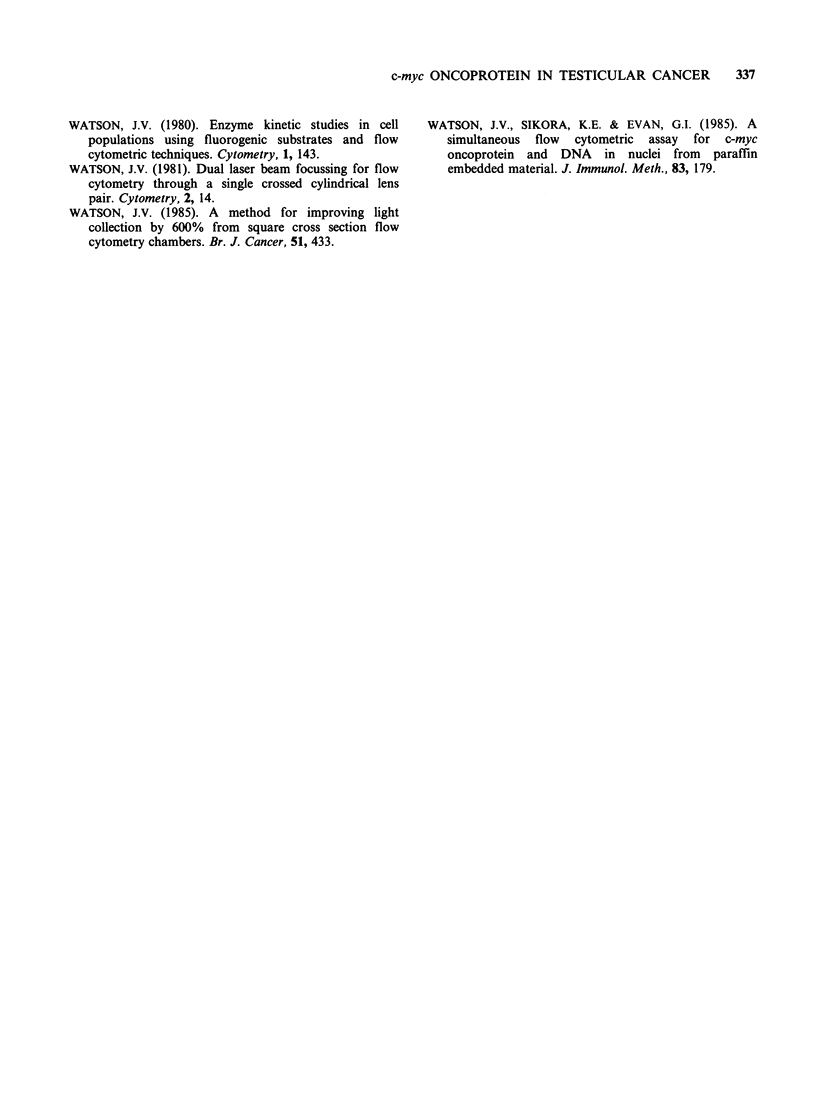# The clinical significance of flow cytometric c-myc oncoprotein quantitation in testicular cancer.

**DOI:** 10.1038/bjc.1986.56

**Published:** 1986-03

**Authors:** J. V. Watson, J. Stewart, G. I. Evan, A. Ritson, K. Sikora

## Abstract

A sensitive flow cytometric assay has been developed using a monoclonal antibody, Myc 1-6E10, to quantitate c-myc oncoprotein levels in nuclei isolated from wax embedded testicular tumours. The oncoprotein (p62c-myc) level increased significantly with increasing teratoma differentiation. Patients with intermediate and undifferentiated tumours who developed recurrence had lower p62c-myc levels than those who were disease free since their initial treatment. Such quantitative biochemical methods may provide new prognostic indices for cancer patients.


*Br. J. Cancer* (1986), **53**, 331–337

### The clinical significance of flow cytometric c-*myc* oncoprotein quantitation in testicular cancer

J.V. Watson[1], J. Stewart[1], G.I. Evan[2], A. Ritson[2] & K. Sikora[2]

[1]*MRC Clinical Oncology Unit;* [2]*Ludwig Institute for Cancer Research, The Medical School, Hills Road, Cambridge CB2 2QH, UK.*

A sensitive flow cytometric assay has been developed using a monoclonal antibody, Myc 1-6E10, to quantitate c-*myc* oncoprotein levels in nuclei isolated from wax embedded testicular tumours. The oncoprotein ($p62^{c\text{-}myc}$) level increased significantly with increasing teratoma differentiation. Patients with intermediate and undifferentiated tumours who developed recurrence had lower $p62^{c\text{-}myc}$ levels than those who were disease free since their initial treatment. Such quantitative biochemical methods may provide new prognostic indices for cancer patients.

Oncogenes are highly conserved regions of the normal genome and over 25 have been identified and many have also been cloned and sequenced (Bishop, 1984; Hamlyn & Sikora, 1983). Changes in either the coding or control regions of these genes have been implicated in the development of cancer (Cooper & Lane, 1984; Krontiris, 1983). Several molecular mechanisms resulting in the increased production of normal oncogene products or the development of aberrant proteins which subvert the normal growth control processes have now been uncovered (Der & Cooper, 1983; Stewart *et al.*, 1984). These include gene amplification, translocation, mutation, promotor insertion and rearrangement. Such changes have been documented in fresh tumour biopsies from patients as well as cultured cell lines. (Rothberg *et al.*, 1984; Favera *et al.*, 1982). The N-*myc* gene has been found to be amplified up to 100-fold in both neuroblastoma (Schwab *et al.*, 1983) and retinoblastoma (Lee *et al.*, 1984) using DNA hybridization techniques. In one patient with chronic myelocytic leukaemia, the c-*myc* sequence was amplified 16-fold and rearranged within the genome during episodes of transformation (McCarthy *et al.*, 1984). Amplification of this gene has been reported in cell lines derived from a very poor prognosis group of patients with small cell lung cancer (Little *et al.*, 1983). K-*ras* and H-*ras* mRNA have been found elevated in colonic carcinoma, colonic polyps (Spandidos & Kerr, 1984) and breast cancer (Spandidos & Agnantis, 1984). The structure and function of the oncogene protein products are now under active investigation. Those of c-*sis* and v-*erb*-B respectively code for a subunit of platelet derived growth factor, PDGF (Waterfield *et al.*, 1983) and the internal domain of the epidermal growth factor receptor (Downward *et al.*, 1984).

Correspondence: J.V. Watson.
Received 9 September 1985; and in revised form, 11 November 1985.

Considerable interest has surrounded the *ras* and *myc* genes as marked variation has been found in the quantity of their transcripts in clinical biopsies at the RNA level (Slamon *et al.*, 1984). The c-*myc* gene product is a 62,000 mol. w protein ($p62^{c\text{-}myc}$) which may be involved in the transition from a quiescent to an actively dividing state. The level of c-*myc* mRNA increases as cells are stimulated into division (Kelly *et al.*, 1983; Rabbitts *et al.*, 1985; Makino *et al.*, 1984; Thompson *et al.*, 1985; Greenberg & Ziff, 1984). Both mRNA transcripts and the protein itself have unusually short half-lives of 20 to 30 min (Rabbitts *et al.*, 1985; Hann *et al.*, 1985), a prerequisite for their putative cell proliferation control function. However, when cells have entered the cell cycle and protein level remains constant in $G_1$, S and $G_2$ (Hann *et al.*, 1985; Rabbitts *et al.*, 1985). Furthermore, the protein binds to the nucleus, one of the likely sites for proliferation control (Evan & Hancock, 1985).

DNA and RNA hybridisation analysis is difficult to perform with many clinical samples. Low copy number genes and message cannot be detected with current methods. Hybridisation techniques cannot normally be applied to fixed embedded material stored in pathology departments. Furthermore, they tell us nothing about the ultimate concentration and distribution in the cell of the final gene product, the oncoprotein. In order to examine the relevance of c-*myc* in clinical samples a set of mouse monoclonal antibodies was constructed against the c-*myc* protein (Evan *et al.*, 1985). One of these antibodies (Myc-16E10) has been used to localize $p62^{c\text{-}myc}$ in formalin fixed histological sections from patients with testicular cancer (Sikora *et al.*, 1985). In this report we show that flow cytometric quantitation of $p62^{c\text{-}myc}$ using Myc 1-6E10 in nuclei extracted from paraffin embedded

© The Macmillan Press Ltd., 1986

testicular cancer biopsies can give prognostic information.

### Materials and methods

### *Production of the anti-p-62$^{c\text{-myc}}$ antibody*

Full details for the production and characterisation of the antibody are given elsewhere (Evan *et al.*, 1985). Briefly, the DNA base sequence of the cloned gene was used to determine the amino acid structure of the protein. Synthetic peptides were then constructed to regions predicted to be hydrophillic and hence likely to be exposed on the surface of the complete molecule. Mice were immunised with the peptides to produce a number of monoclonal antibodies (MCAs) which immunoprecipitate a 62,000 dalton protein identifiable with the c-*myc* product. (Evan *et al.*, 1985). One of these antibodies, Myc 1-6E10 raised to the 18 amino acid sequence from residues 173–188 (D-peptide), has been demonstrated to detect p62$^{c\text{-}myc}$ in nuclei extracted from archival material (Watson *et al.*, 1985).

### *Patients*

A total of 38 patients attending Addenbrooke's Hospital with testicular cancer between the years 1967 to 1982 were included in this study. The only criteria of entry were a minimum follow up of 3 years from diagnosis and that sufficient material could be obtained from the stored biopsy of the primary lesion to perform the assay.

### *Extraction of nuclei, staining and flow cytometry*

The full technical details of our method have been published (Watson *et al.*, 1985), but for convenience, the methodology is summarised in Figure 1. The paraffin wax embedded biopsies were obtained from the Department of Pathology and care was taken, where possible, to check that the 20 $\mu$m sections used for analysis contained a majority of tumour tissue. This was effected by cutting alternate 4 $\mu$m and 20 $\mu$m sections and examining the former histologically. Sufficient biopsy material was available from 33 of the 38 patients to perform this check and the vast majority of each section analysed was composed of tumour. This is consistent with the clinical behaviour of these lesions which tend to have infiltrated the majority of the testicular tissue at diagnosis. Isolated nuclei were extracted by pepsin digestion after dewaxing and xylene and rehydration. Our procedure is a modified version of the method described by Headley *et al.*, (1983). The suspension containing isolated nuclei was filtered through a 35 $\mu$m nylon mesh to remove debris and clumps, centrifuged at 200 *g* and resuspended in PBS, pH 7.4 at a concentration of $10^6$ nuclei ml$^{-1}$.

Aliquots of 1.0 ml of the nuclear suspension were then placed into 1.5 ml tubes and spun down in an Eppendorf centrifuge. The supernatants were carefully removed and the nuclear pellets were resuspended in dilutions of Myc 1-6E10. A fluorescein labelled rabbit and anti-mouse immunoglobulin was added to probe the Myc 1-6E10 and the nuclei were counterstained with propidium iodide (PI) to assess DNA content (Watson *et al.*, 1985).

The nuclei were analysed in the Cambridge MRC custom built flow cytometer (Watson, 1980, 1981) with a high efficiency light collection flow chamber (Watson, 1985). The Innova-90 argon laser (Coherent, Palo Alto, CA) was turned to the 488 nm line which simultaneously excites red (DNA) and green (oncoprotein) fluorescence from individual nuclei. The data were stored on computer disc and following collection were recalled for display and analysis. Figure 2 shows an example of a data set from one patient with teratoma where a well pronounced aneuploid

**Figure 1** Schema for nuclear p62$^{c\text{-}myc}$ assay from wax block material.

**Figure 2** Example of flow cytometric analysis of $p62^{c\text{-}myc}$ (ordinate) *versus* total DNA content (abscissa) from a patient with aneuploid tumour elements. The first DNA peak corresponds in position to the normal diploid which is associated with low $p62^{c\text{-}myc}$ levels. The aneuploid peak is associated with high levels.

component was present. The data are presented as contour plots of $p62^{c\text{-}myc}$ associated fluorescence (ordinate) *versus* DNA fluorescence (abscissa). Note the higher oncoprotein fluorescence associated with the aneuploid peak. These data were obtained from a very small biopsy of the epididymis which was infiltrated by tumour. The whole of the embedded specimen was used for the analysis and we can only infer that the diploid component (35% of the total) was normal tissue.

### Results

Specificity controls for antibody binding and signal generation were performed. Blocking assays were carried out with two peptides. The D-peptide, immunogen for Myc 1-6E10, and the G-peptide corresponding to the 32 amino acid carboxy terminus of $p62^{c\text{-}myc}$ (residues 408–439) were used. Two $\mu$l of the peptide solutions ($1\,mg\,ml^{-1}$) were each added to $20\,\mu l$ antibody ($2\,mg\,ml^{-1}$) before use in the assay, the peptide concentrations being in considerable excess. The D-peptide completely blocked antibody binding activity on seminoma, teratoma and two tissue culture cell lines. The G-peptide caused only a 14% and 11% decrease in the Myc 1-6E10 signal in seminoma and teratoma respectively. These results, which indicate specificity, are shown in Table I. Four monoclonal antibodies which do not recognise $p62^{c\text{-}myc}$ or nuclear structures gave no significant signal above background in the two tissue culture lines.

**Table I** Results of blocking assay with the D and G peptides

| | *Seminoma* | *Teratoma* |
|---|---|---|
| FITC Control | 93 | 136 |
| Myc 1-6E10 | 489 | 426 |
| D-peptide blocked | 78 | 102 |
| G-peptide blocked | 421 | 380 |

The median fluorescence values associated with the diploid peak in seminoma and the aneuploid peak from a teratoma are shown for unblocked Myc 1-6E10 and after addition of the D-peptide (used as the immunogen) and the G-peptide (corresponding to the carboxy terminus of $p62^{c\text{-}myc}$) prior to the assay. Binding was blocked completely by the D-peptide but the G-peptide caused only a 14% and 11% signal decrease in seminoma and teratoma respectively.

An overall summary of $p62^{c\text{-}myc}$ quantitation in testicular cancer *versus* clinical outcome is given in Figure 3. All assays were performed blind and the median of the $p62^{c\text{-}myc}$ fluorescence distribution associated with the diploid peak or the aneuploid component when present (3 patients) was calculated for each sample. The patients were then divided into two groups, those who were alive and well with no recurrence at 3 years after diagnosis and those who developed recurrence within this interval. The mean oncoprotein levels in arbitrary fluorescence units ($\pm$s.d.), were $513\pm275$ and $155\pm77$ for the good and bad prognosis groups respectively. Student's $t$-test for comparison of the means with their variance was 3.4 with 36 degrees of freedom, $P<0.001$. The good prognosis group contained 11 patients with seminoma (open circles Figure 3) all of whom are alive and well. When this subset was excluded the mean $p62^{c\text{-}myc}$ level was $456\pm305$ for the teratoma patients who are alive and well. Comparison of this group with those who developed recurrence gave $t=2.4$ and 25 degrees of freedom, $P<0.02$.

Comparisons of histological type with $p62^{c\text{-}myc}$ are shown in Figure 4. The mean level from the 11 patients with seminoma was $601\pm204$. Twenty-seven patients with teratoma were classified into 3 groups: undifferentiated (MTU, 16 patients); intermediate (MTI, 6 patients) and tumours of intermediate differentiation with yolk sac elements (MT+YS, 5 patients). The mean $p62^{c\text{-}myc}$ levels were $185\pm119$, $340\pm142$ and $862\pm118$ for MTU, MTI and MT+YS respectively. The mean values

**Figure 3** Comparison of p62$^{c\text{-}myc}$ content in nuclei from biopsies of good and bad prognosis testicular cancer patients. A/W = alive and well; R/D = recurrence and dead.

**Figure 4** p62$^{c\text{-}myc}$ content of tumour biopsy nuclei from patients with seminoma (sem); malignant teratoma undifferentiated (MTU); malignant teratoma intermediate (MTI); malignant teratoma with yolk sac elements (MT+YS). Differences at the $P<0.05$ level of significance were found for comparisons between seminoma and MTI, seminoma and MT+YS and between MTI and MTU. Differences between seminoma and MTU and between MTI and MT+YS were significant at $P<0.005$, and between MTU and MT+YS at $P<0.0001$.

were statistically different for all comparisons at $P<0.05$ (see legend Figure 4).

Nine patients with MTU and 1 patient with MTI died or developed recurrent disease within three years of diagnosis, the remaining 12 patients have remained alive and well with no recurrence for at least 3 years. When the MTU and MTI categories were combined we found the mean p62$^{c\text{-}myc}$ levels in the good and bad prognosis groups were $288 \pm 157$ and $155 \pm 77$ respectively (Figure 5). *t*-test for comparison of means was 1.78 with 20 degrees of freedom, $P<0.05$.

There was no correlation between p62$^{c\text{-}myc}$ level and the age of the patient, stage of disease, or serum levels of human chorionic gonadotrophin. The pre-treatment alpha foetoprotein levels were compared with p62$^{c\text{-}myc}$ and there was no correlation in MTU or MTI but a good correlation with tumours showing yolk sac differentiation.

Only 3 patients exhibited an aneuploid component on the DNA histogram. All were teratomas but the numbers are too small to make any useful observations on the prognostic significance of aneuploidy in this group of patients. However, these data sets gave some indication of p62$^{c\text{-}myc}$ levels in normal tissue assuming that the diploid components were indeed normal. The diploid peak in Figure 2 had a median p62$^{c\text{-}myc}$ level of 132 after subtracting the corresponding fluorescence control value. In the remaining two patients the diploid component comprised 10% and 30% of cells in the sample. These proportions correlated with the subjective assessment of normal cell content in the 4 $\mu$m histological sections. The p62$^{c\text{-}myc}$ levels in these diploid peaks were 68 and 110 respectively. One further patient, not included in this study, showed haploid, diploid and aneuploid components. This clearly was an 'odd-ball' from the DNA histogram and was later found to be an interstitial cell tumour. Both the haploid

**Figure 5** Comparison of p62$^{c\text{-}myc}$ content in nuclei from biopsies of good and bad prognosis teratoma patients after exclusion of the best prognosis group, those showing yolk sac differentiation. A/W = alive and well; R/D = recurrence and dead.

and diploid peaks had low p62$^{c\text{-}myc}$ fluorescence, 50 and 90 respectively.

### Discussion

We believe this to be the first report of an oncogene product quantitation in archival biopsies which has been correlated with morphology and clinical prognosis. Significant differences were found for all comparisons between seminoma and the three histological categories of teratoma. The p62$^{c\text{-}myc}$ level increased with increasing differentiation in teratoma, and the good prognosis patients had significantly higher levels than the bad prognosis group. Presumed normal elements in biopsies containing aneuploid components had low p62$^{c\text{-}myc}$ fluorescence with a mean of 90. This corresponds to the lowest end of the range found in MTU (Figure 3). Completely normal control testicular tissue within the age group 15 to 55 years spanning the ages of patients in this study was, comfortingly, impossible to find in our pathology archives. Orchidectomy specimens from patients with prostatic cancer were plentiful but the majority of these patients were over 65 and had been treated with oestrogens. Histologically these specimens were atrophic and totally unsuitable as controls for the patients reported here.

The findings in teratoma were completely contrary to our initial expectation as oncogene amplification or increased mRNA transcripts have been found in a number of malignancies (Schwab *et al.*, 1983; Lee *et al.*, 1984; McCarthy *et al.*, 1984; Little *et al.*, 1983; Spandidos & Kerr, 1984; Spandidos & Agnantis, 1984). However, an increase in either the gene or mRNA copy number (or both), which should give rise to an increased protein production rate, need not necessarily be reflected in a marked increase in the total protein content for two main reasons. Firstly, inappropriately increased message may result in rate limitation at the protein synthesis level. Secondly, an increase in protein degradation may offset an increased production rate. The latter is most likely to occur with a protein which has a short half-life and clearly, this is a distinct possibility for p62$^{c\text{-}myc}$ with a half-life of 20–30 min in rapidly cycling and stimulated cells (Rabbitts *et al.*, 1985; Greenberg & Ziff, 1984). Hence, the lower absolute levels in undifferentiated teratomas, compared with the better differentiated tumours, may reflect an increased protein turnover and an increased cell production rate in the former. A further possibility is that post transcriptional protein modification in the more malignant tumours may give rise to an alteration or partial occlusion of the epitope recognised by Myc 1-6E10. However, these possibilities will not be resolved with archival data and will probably require the function of the protein to be elucidated.

In spite of our lack of knowledge of the protein's function these results demonstrate that there is considerable potential clinical significance in quantitating oncogene products in wax embedded stored biopsy material. The clinical outcome will already be known in most cases and consequently prognostic correlates can be discovered rapidly. Our results using Myc 1-6E10 with immunoperoxidase staining in teratoma show good agreement between the quantitative flow cytometric analysis and the subjective results of immunohistology (Sikora *et al.*, 1985).

One of our objectives in these studies was to define quantitative biochemical differences between good and bad prognosis groups in which the morphology is very similar or identical. This would be of direct relevance to patient care by being able to identify those poor risk patients for whom new therapeutic schedules would have to be found. These studies have gone some way to achieving this by demonstrating a difference between the mean p62$^{c\text{-}myc}$ levels in the good and bad prognosis teratoma groups. Although we have found significant differences between the prognostic subsets in teratoma it would not be possible to predict reliably into which group an individual would fall from our current data (see Figure 5). However, c-*myc* is one of many oncogenes and the measurement of combinations of these gene products simultaneously may provide more accurate prognostic data. Those of the p53, c-*fos* and c-*myb* genes are also nuclear binding and should, therefore, be amenable to analysis using this method when suitable MCAs become available.

JS holds a Cancer Research Campaign research fellowship. We thank Professor J.M. Bishop and A. Gresham, and Drs S. Brenner and D. Wight for helpful discussion. We are indebted to S. Insole, L. Barnes, N. Grant and K. Harvey for the artwork.

### Reference

BISHOP, J.M. (1984). Cancer genes come of age. *Cell*, **32**, 1018.

COOPER, G.M. & LANE, M.A. (1984). Cellular transforming genes and oncogenesis. *Biochem. Biophys. Acta*, **734**, 9.

DER, C.J. & COOPER, G.M. (1983). Altered gene products are associated with activation of cellular *ras*$^{K}$ Genes in human lung and colon carcinomas. *Cell*, **32**, 201.

DOWNWARD, J., YARDEM, Y., MAYES, E. & 6 others. (1984). Close similarities of epidermal growth factor receptor and v-*erb* B oncogene protein sequences. *Nature*, **307**, 521.

EVAN, G. & HANCOCK, D. (1985). Studies on the interaction of the human c-*myc* protein with cell nuclei: p62$^{c\text{-}myc}$ as a member of a discrete subset of nuclear proteins. *Cell*, **43**, 253.

EVAN, G., LEWIS, G.K., RAMSAY, G. & BISHOP, J.M. (1985). Isolation of monoclonal antibodies specific for human and mouse proto-oncogene products. *Mol. Cell Biol.*, **5**, 3610.

FAVERA, R.D., WONG-STAAL, F. & GALLO, R. (1982). *Onc* gene amplification in promyelocytic leukaemia cell line HL-60 and primary leukaemic cells of the same patient. *Nature*, **299**, 61.

GREENBERG, M.E. & ZIFF, E.B. (1984). Stimulation of 3T3 cells induces transcription of the c-*fos* proto-oncogene. *Nature*, **311**, 433.

HAMLYN, P.H. & SIKORA, K. (1983). Oncogenes. *Lancet*, **ii**, 326.

HANN, S.R., THOMPSON, C.B. & EISENMAN, R.N. (1985). c-*myc* oncogene protein is independent of the cell cycle in human and avian cells. *Nature*, **314**, 366.

HEADLEY, D. W., FRIEDLANDER, M.I., TAYLOR, I.W., RUGG, C.A. & MUSGROVE, E.A. (1983). Method for analysis of cellular DNA content of paraffin-embedded pathological material using flow cytometry. *J. Histochem. Cytochem.*, **31**, 1333.

KELLY, K., COCHRAN, B.H., STILES, C.D. & LEDER, P. (1983). Cell specific regulation of the c-*myc* gene by lymphocyte mitogens and platelet derived growth factor. *Cell*, **35**, 603.

KRONTIRIS, T.G. (1983). The emerging genetics of human cancer. *New Engl. J. Med.*, **309**, 404.

LEE, W.W., MURPHEE, A.L. & BENEDICT, W.F. (1984). Expression and amplification of the N-*myc* gene in primary retinoblastoma. *Nature*, **309**, 458.

LITTLE, C.D., NAU, M.M., CARNEY, D.N., GAZDAR, A.F. & MINNA, J.D. (1983). Amplification and expression of the c-*myc* oncogene in human lung cancer cell lines. *Nature*, **306**, 194.

MAKINO, R., HAYASHI, K.A. & SUGIMURA, T. (1984). c-*myc* is induced in rat liver at a very early stage of regeneration or by cycloheximide treatment. *Nature*, **310**, 697.

McCARTHY, D.M., RASSOOL, F.V., GOLDMAN, J.M., GRAHAN, S.V. & BIRNIE, G.D. (1984). Genomic alterations involving the c-*myc* proto-oncogene locus during the evolution of a case of chronic granulocytic leukaemia. *Lancet* **ii**, 1362.

RABBITTS, P.H., WATSON, J.V., LAMOND, A. & 7 others. (1985). Metabolism of c-*myc* gene products: c-*myc* mRNA and protein expression in the cell cycle. *Embo J.*, **4**, 2009.

ROTHBERG, P.G., ERISMAN, M.D., DIEHL, R.E., ROVIATTI, U.G. & ASTRIN, S.M. (1984). Structure and expression of the oncogene c-*myc* in fresh tumour material from patients with haemopoetic malignancies. *Mol. Cell Biol.*, **4**, 1096.

SCHWAB, M., ALITALO, K., KLEMPNAUER, K.-H. & 6 others. (1983). An amplified gene with limited homology to *myc* cellular oncogene is shared by human neuroblastoma cell lines and a neuroblastoma tumour. *Nature*, **305**, 245.

SIKORA, K., EVAN, G., STEWART, J. & WATSON, J.V. (1985). Detection of c-*myc* oncogene product in testicular cancer. *Br. J. Cancer*, **52**, 171.

SLAMON, D.J., DEKERNION, J.B., VERMA, I.M. & CLINE, M.J. (1984). Expression of cellular oncogenes in human malignancies. *Science*, **224**, 256.

SPANDIDOS, D.A. & AGNANTIS, N.J. (1984). Human malignant tumours of the breast, as compared to their respective normal tissue, have elevated expression of the Harvey *ras* oncogene. *Anticancer Res.*, **4**, 269.

SPANDIDOS, D.A. & KERR, I.B. (1984). Elevated expression of the human *ras* oncogene family in premalignant tumours of the colorectum. *Br. J. Cancer*, **49**, 681.

STEWART, T.A., PATTENGALE, P.K. & LEDER, P. (1984). Spontaneous mammary adenocarcinomas in transgenic mice carry and express MTV/myc fusion genes. *Cell*, **38**, 627.

THOMPSON, C.B., CHALLONER, P.B., NEIMAN, P.E. & GROUDINE, M. (1985). Levels of c-*myc* oncogene mRNA are invariant throughout the cell cycle. *Nature*, **314**, 363.

WATERFIELD, M.D., SCRACE, G.T., WHITTLE, N. & 6 others. (1983). Platelet derived growth factor is structurally related to the putative transforming protein p28$^{sis}$ of simian sarcoma virus. *Nature*, **304**, 35.

WATSON, J.V. (1980). Enzyme kinetic studies in cell populations using fluorogenic substrates and flow cytometric techniques. *Cytometry*, **1**, 143.

WATSON, J.V. (1981). Dual laser beam focussing for flow cytometry through a single crossed cylindrical lens pair. *Cytometry*, **2**, 14.

WATSON, J.V. (1985). A method for improving light collection by 600% from square cross section flow cytometry chambers. *Br. J. Cancer*, **51**, 433.

WATSON, J.V., SIKORA, K.E. & EVAN, G.I. (1985). A simultaneous flow cytometric assay for c-*myc* oncoprotein and DNA in nuclei from paraffin embedded material. *J. Immunol. Meth.*, **83**, 179.